# Overexpression of Wilms Tumor 1 Gene as a Negative Prognostic Indicator in Acute Myeloid Leukemia

**DOI:** 10.1371/journal.pone.0092470

**Published:** 2014-03-25

**Authors:** Xiaodong Lyu, Yaping Xin, Ruihua Mi, Jing Ding, Xianwei Wang, Jieying Hu, Ruihua Fan, Xudong Wei, Yongping Song, Richard Y. Zhao

**Affiliations:** 1 Henan Institute of Hematology, Affiliated Tumor Hospital of Zhengzhou University, Zhengzhou, Henan, China; 2 Department of Endocrinology and Metabolic Diseases, the Second Affiliated Hospital of Zhengzhou University, Zhengzhou, Henan, China; 3 Division of Molecular Pathology, Department of Pathology, University of Maryland School of Medicine, Baltimore, Maryland, United States of America; George Mason University, United States of America

## Abstract

Chromosomal aberrations are useful in assessing treatment options and clinical outcomes of acute myeloid leukemia (AML) patients. However, 40∼50% of the AML patients showed no chromosomal abnormalities, *i.e*., with normal cytogenetics aka the CN-AML patients. Testing of molecular aberrations such as *FLT3* or *NPM1* can help to define clinical outcomes in the CN-AML patients but with various successes. Goal of this study was to test the possibility of Wilms’ tumor 1 (*WT1*) gene overexpression as an additional molecular biomarker. A total of 103 CN-AML patients, among which 28% had overexpressed *WT1*, were studied over a period of 38 months. Patient’s response to induction chemotherapy as measured by the complete remission (CR) rate, disease-free survival (DFS) and overall survival (OS) were measured. Our data suggested that *WT1* overexpression correlated negatively with the CR rate, DFS and OS. Consistent with previous reports, CN-AML patients can be divided into three different risk subgroups based on the status of known molecular abnormalities, i.e., the favorable (*NPM1^mt^*/no *FLT3^ITD^*), the unfavorable (*FLT3^ITD^*) and the intermediate risk subgroups. The *WT1* overexpression significantly reduced the CR, DFS and OS in both the favorable and unfavorable groups. As the results, patients with normal *WT1* gene expression in the favorable risk group showed the best clinical outcomes and all survived with complete remission and disease-free survival over the 37 month study period; in contrast, patients with *WT1* overexpression in the unfavorable risk group displayed the worst clinical outcomes. *WT1* overexpression by itself is an independent and negative indicator for predicting CR rate, DFS and OS of the CN-AML patients; moreover, it increases the statistical power of predicting the same clinical outcomes when it is combined with the *NPM1* ^mt^ or the *FLT3* ^ITD^ genotypes that are the good or poor prognostic markers of CN-AML.

## Introduction

Acute myeloid leukemia (AML) is defined as hematopoietic stem cell malignancy characterized by clonal expansion of myeloid blasts. It is typically divided into three different risk groups, *i.e*, the favorable, the intermediate and the unfavorable group based on the types of chromosomal aberrations. About half (40∼50%) of the AML patients have normal karyotype or normal cytogenetics that typically belong to the intermediate risk group in terms of patient’s survival [1], [2]. However, inconsistencies were found among this group of patients in their responses to chemotherapy and prognosis that sometimes makes it difficult to make the right decision for therapeutic treatment and/or assessment of the possible treatment outcome of the patients.

Adding examination of molecular aberrations is thought to be helpful in addressing the differences as described above. Few molecular markers have been used to predict treatment response and prognosis in cytogenetically normal acute myeloid leukemia (CN-AML), such as the nucleoplasmin (*NPM1*) gene and the fms-like tyrosine kinase 3 (*FLT3*) gene. A typical *NPM1* gene mutation includes small insertions (4∼11 bp) in the coding region of exon 12. The *FLT3* gene mutations usually include a D835 point mutation in the tyrosine kinase domain (TKD) of the exon 20 or internal tandem duplications (ITD) in the exon of 14 or 15. Detection of these *NPM1* and *FLT3* gene mutations has been used to evaluate clinically biological behavior of leukemia cell in the CN-AML patients [2], [3].

The Wilms’ tumor 1 (*WT1*) gene, which is located on the chromosome 11p13, encodes a zinc-finger transcriptional factor that has emerged as an important regulator of normal and malignant hematopoiesis. *WT1* is also one of the molecules that are known to control cellular apoptosis [4]. Resistance of leukemia blasts to apoptosis may cause poor clinical outcomes. Therefore, regulation of apoptotic or anti-apoptotic pathways has high clinical relevance with regard to the remission therapy and the overall survival of the AML patients. Interestingly, high *WT1* gene expression was consistently found in peripheral blood (PB) or bone marrow (BM) in the AML patients in comparison with normal controls [5]. However, the significance of *WT1* overexpression in therapeutic response and prognosis are still elusive in CN-AML [6]–[8]. Goal of this study was to examine possible correlation of *WT1* gene expression with therapeutic response and prognosis in the CN-AML patients. In addition, we also examined the possible interactions of *WT1* gene overexpression with the *NPM1* or *FLT3* gene status, which are known molecular markers associated with the survival and treatment outcomes of AML patients.

## Materials and Methods

### Patient Population

A total of 103 CN-AML patients consisting of 58 males and 45 females with a median age of 42 years (range, 17–82 years) were recruited for the *WT1* overexpression study. All patients were newly diagnosed patients with CN-AML at the Henan Cancer Hospital from the time period of September of 2009 to October, 2012, *i.e.*, a total of 38 months. The diagnosis of AML was made according to the FAB classification. The M3 patients were not included in this study because of the success in chemotherapy based on all-trans-retinoic acid and arsenic trioxide. The standard RHG banding techniques were employed in the karyotyping of leukemia. One milliliter of bone marrow was collected in the EDTA vacutainer from all 103 patients before treatment. This clinical study was approved by the Committee of International and Scientific Research at the Affiliated Tumor Hospital of Zhengzhou University. Written informed consents were obtained from all patients for this study. If a minor was enrolled in this study, a letter of authorization will be first obtained from minor’s guardian along with a signed informed consent by the guardian.

The *WT1* overexpression was defined in this study as ≥250 copies/10^4^ *ABL* as previously recommended [9]. When the *WT1* gene was not detected by real-time PCR or the copy number was under the lower limit of detection (3×102 copies/mL), we denoted this *WT1* gene copy number as “non-detectable” or “ND”. Based on this criterion, the *WT1* gene overexpression was detected in 29 of 103 patients (28%) with a median value of 720 copies/10^4^ *ABL* (ranged from ND to 8.2×10^6^ copies/mL).

Among these patients, 29% (30/103) of them carried 3 different *NPM1* ^mt^ mutant genotypes. The most common *NPM1* ^mt^ mutation was the type A (80%, 24/30), which had a “TCTG” insertion in exon 12. In addition, 4 of 30 patients (13%) had the type B mutation (“CATG” insertion), and 2 of 30 patients (7%) had the type 13 mutation (“TAAG” insertion) [10]. Seven of the 30 *NPM1* ^mt^ carrying patients (23%) also showed high *WT1* gene expression.

The *FLT3* mutation was detected in 27% of the total patient cohort (28/103), which included the *FLT3* ^TKD^ and *FLT3* ^ITD^ mutations, respectively. In which 10 out of the 28 patients (36%) carried the *FLT3* ^TKD^ mutation including the D835H (80%, 8/10), D835V (10%, 1/10) and D835Y (10%, 1/10) point mutations, respectively. The *FLT3* ^ITD^, which is generally associated with unfavorable outcome [2], [3], was identified in 18 of the 28 patients (64%) that carried the internal tandem duplication in exon 14 (61%, 11/18), intron 14 (6%, 1/18) and exon 15 (33%, 6/18). As shown in **Table 1**, 30% (3/10) of the *FLT3* ^TKD^–carrying patients and 44% (8/18) of the *FLT3* ^ITD^–carrying patients also showed *WT1* gene overexpression.

**Table 1:** Correlation of WT1 overexpression with clinical data, FAB subtypes, and molecular abnormalities in CN-AML patients.

Variant	Total (N = 103)	WT1^op^ (N = 29, 28%)	*P*
Median age, years (range)	42 (17∼82)	44 (29∼74)	.*809*
**Age in groups, years**			.*549*
≤60	91	27 (30)	
>60	12	2 (17)	
**Sex**			.*238*
Male	58	19 (33)	
Female	45	10 (22)	
**WBC count, 10^9^/L**			.*906*
20 or below	40	11 (28)	
Above 20	63	18 (29)	
**FAB subtype**			
M0	4	2 (50)	.*672*
M1	16	3 (19)	.*543*
M2	41	13 (32)	.*515*
M4	16	4 (25)	.*998*
M5	26	7 (27)	.*872*
**Molecular abnormalities**			
NPM1^mt^	30	7 (23)	.*485*
FLT3^TKD^	10	3 (30)	*1*
FLT3^ITD^	18	8 (44)	.*091*
**Risk molecular subgroups** *			
**Favorable**			
NPM1^mt^/no FLT3^ITD^	23	4 (17)	.*193*
**Unfavorable**			
FLT3^ITD^	18	8 (44)	.*091*
**Intermediate**			
Others excluding NPM1^mt^/no FLT3^ITD^ and FLT3^ITD^	62	17 (27)	.*838*

WT1^op^, WT1 overexpression; FAB, French-American-British; CN-AML, cytogenetically normal acute myeloid leukemia; WBC, white blood cell.

*stratification based on molecular abnormalities [2].

### Treatment

All patients received one or two courses of induction chemotherapy with DA (daunorubicin 45 mg/m^2^×3 days; cytarabine 100–200 mg/m^2^ every 12 hours×7 days) or HA (harringtonine 4–6 mg/m^2^×7 days; cytarabine 100–200 mg/m^2^ every 12 hours ×7 days). If patients get complete remission, they would receive consolidation chemotherapy with high-dose cytarabine (3 g/m^2^ every 12 hours on days 1, 3, 5 and 7 for a total of 24 g/m^2^). Otherwise they would continue to receive other induction chemotherapies. Whether patient will receive hematopoietic stem cell transplantation (HSCT) will depend on comprehensive clinic situation.

### DNA and RNA Extraction

Genomic DNA was extracted by using the Genomic DNA Extraction Kit (Tiangen, Beijing, China). Total RNA was isolated using the Trizol reagent (Invitrogen, Carlsbad, USA). All protocols were conducted according to manufacturer’s instructions. The quality and concentration of DNA and RNA were analyzed with a Biophotometer (Eppendorf AG, Hamburg, Germany).

### Quantification of *WT1* Gene Expression

Real-time reverse-transcriptase polymerase chain reactions (RT-PCR) using patient-derived RNA were carried out on the 7300 real-time PCR system (Applied Biosystems, Foster City, CA, USA). Commercially available *WT1* mRNA quantification kit (Yuanqi, Shanghai, China) was used to detect *WT1* gene expression. A housekeeping gene *ABL* was used as an internal control for calibration of possible variations caused by the variable efficiencies of RNA extraction, RT-PCR and operation. The relative levels of the *WT1* expression to the *ABL* control of the clinical samples were calculated by simultaneous reaction with series standards of known concentrations (3×10^3–6^ copies/ml). To calculate copy number of a specific sample, we first established a standard curve by using a series of known commercial standards from 3×10^3^ to 3×10^6^ copies/mL. The linear dynamic range of this method was from 3×10^2^ copies/mL to 3×10^7^ copies/mL. If a samples copy number was above the top limit, the sample will be re-measured with dilutions.

The data were analyzed using the Sequence Detection Software Version 1.2 (Applied Biosystems). For analysis of samples, detectable *WT1* copy numbers were expressed as copies per 10^4^ *ABL* copies according to manufacturer’s instruction. The cutoff for normal *WT1* expression was defined as 250 copies/10^4^ *ABL* as previously used in BM [10].

### Detection of *NPM1* and *FLT3* Gene Mutations

For detection of the *NPM1* and *FLT3* mutations, we carried out PCR gene amplification by using the 9700 PCR amplification system (Applied Biosystems). Exon 12 of the *NPM1* gene and exon 14, 15 and 20 of the *FLT3* gene were amplified using respective primer pairs, *NPM1* ex12-F (5′-TTAACTCTCTGGTGGTAGAATGAA-3′), *NPM1* ex12-R (5′- TGTT ACAGAAATGAAATAAGACGG-3′), *FLT3* ex14-F (5′-TTCCCTTT CATCCAAGAC-3′), *FLT3* ex14-R (5′-AAACATTTGGCACATTCC-3′), *FLT3* ex15-F (5′-GCAATTTAGGTATGAAAGCCAGC-3′), *FLT3* ex15-R (5′-CTTTCAGCATTTTG ACGGCAACC-3′), *FLT3* ex20-F (5′-CCAGGAACGTGCTTGTCA-3′) and *FLT3* ex20-R (5′-TCAAAAA TGCACCACAGTGAG-3′). PCR reaction was performed in a total volume of 25 μL containing 100 ng of DNA, 10 μM of each primer and 12.5 μL 2×PCR buffer (containing MgCl_2_, dNTP mix and Taq polymerase) (Tiangen). PCR reactions were carried out as follows: denaturation at 95°C for 5 minutes, annealing at 55°C for 1 min. and extension at 72°C for 1 min. for 40 cycles. Amplification products were detected by 1% agarose gel electrophoresis. If the PCR product size was correct, amplification products were subsequently confirmed by nucleotide sequencing using the ABI PRISM 3100 genetic analyzer (Applied Biosystems) after purification. The sequencing results were then compared with the reference and wild type sequences of (*NPM1*: GenBank, NG-016018.1) or *FLT3 (*GenBank, NG-007066.1). Gene mutations were confirmed by nucleotide sequencing with coverage of double-strand DNA by using the forward and reverse primers.

### Definitions and Statistical Analysis

Complete remission was characterized by morphologically normal marrow with <5% blasts, neutrophil count >1×10^9^/L, platelet count >100×10^9^/L and normal physical for more than 1 month. The CR rate was evaluated after received one or two courses of induction chemotherapy. Relapse was defined as the reappearance of blasts in the blood or the finding of more than 5% blasts in the bone marrow or any other evidence of leukemia recurrence. The DFS in patients who achieved CR was estimated from the date of CR to relapse or death. The OS was defined as the time from diagnosis to death by any causes [11].

For descriptive statistics, we calculated median, range and percentage of the cases. Proportion was compared using the Chi-Square test. Survival probability was estimated using the Kaplan–Meier curves and the difference between groups was analyzed by the log-rank test. Multivariate analysis was performed applying the COX regression model. For all statistical analyses, the *p* value that was 2-tailed with less than 0.05 was considered to be statistically significant. Statistical analyses were performed by using the statistical software package SPSS Version 19.0 (SPSS Science).

## Results

### Correlation of *WT1* Overexpression with Clinical Parameters

A total of 103 CN-AML patients, consisting of 58 males and 45 females with a median age of 42 years (range, 17–82 years) were recruited to this study. The *WT1* overexpression was defined as ≥250 copies/10^4^ *ABL*. Objective of this part of the study was to examine the possible correlation of the *WT1* gene overexpression with various common clinical features or molecular abnormalities.

Specifically, the *WT1* gene overexpression was detected in 29 of 103 patients (28%) with a median value of 720 copies/10^4^ *ABL* (range: ND to 8.2×10^6^). Comparisons of *WT1* gene overexpression with various clinical parameters and their possible statistical significance are summarized in **Table 1**. Initial comparisons of the *WT1* gene overexpression with common clinical parameters such as age, sex, WBC counts and the FAB subtypes showed no significant differences between the patient groups with or without *WT1* gene overexpression. In order to examine the possible role of *WT1* gene overexpression in assessing treatment response, prognosis and survival of AML patients with normal cytogenetics, All CN-AML patients were divided into three different risk subgroups based on their known molecular abnormalities such as the *FLT3* and *NPM1* gene mutation statuses. As recommended previously [2], [3], the favorable outcome subgroup (n = 23) are those CN-AML patients who have the *NPM1* gene mutation (*NPM1* ^mt^) but do not have the internal tandem duplication in the *FLT3* gene (*FLT3* ^ITD^). The unfavorable outcome subgroup (n = 18) are those patients with the FLT3^ITD^ gene mutations. The remaining patients (n = 62), who do not have the *NPM1* ^mt^/no *FLT3* ^ITD^ nor the *FLT3* ^ITD^ genotypes were defined as the intermediate risk subgroup [2], [3]. No statistical significant differences were found in all of these risk groups. Together, these data suggest that the *WT1* overexpression is not associated with any particular risk group or a clinical parameter.

### Role of *WT1* Overexpression in Response to Induction Chemotherapy

**Figure 1:**
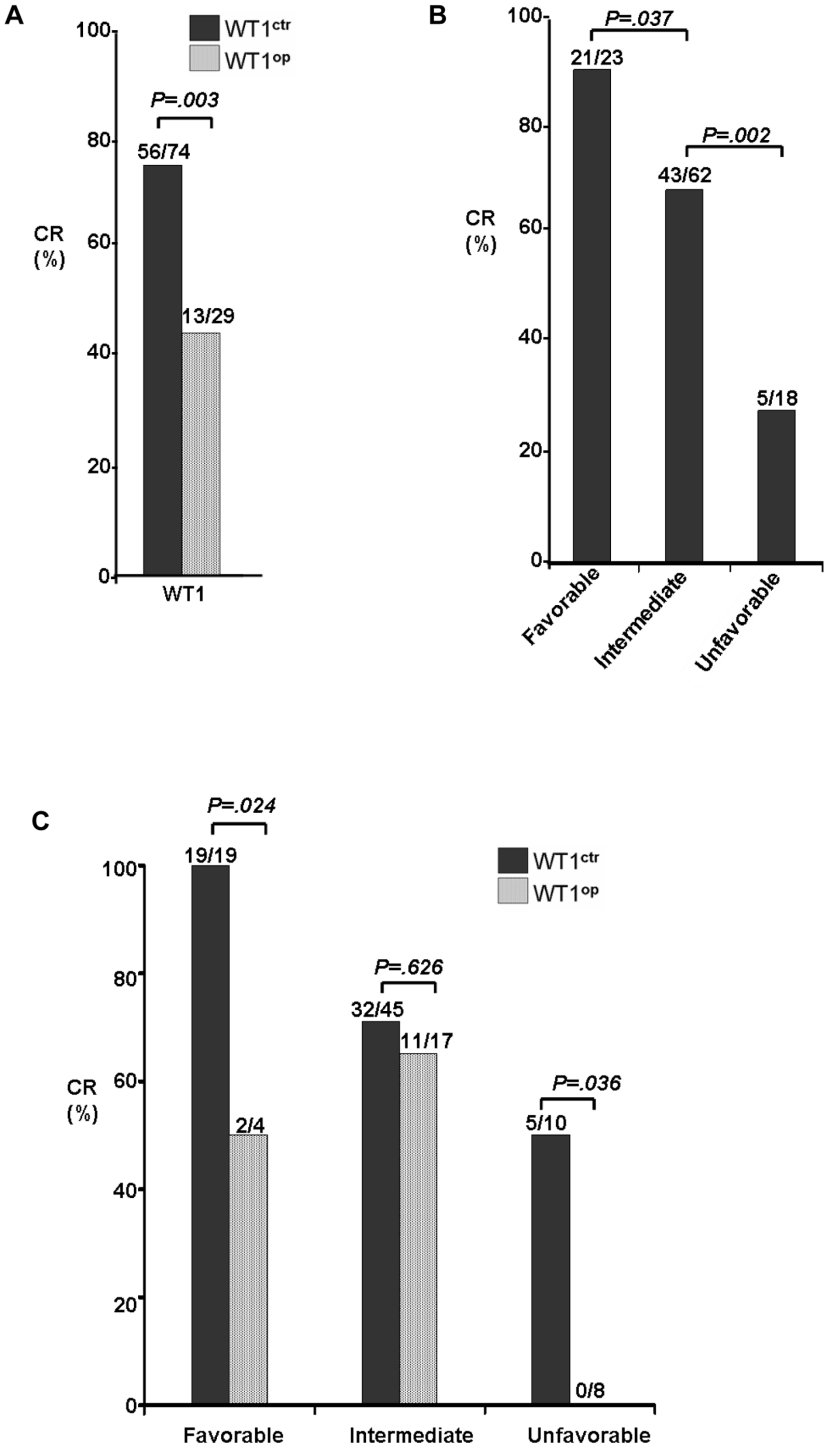
Complete remission (CR) rate analysis based on the *WT1* expression status and other molecular abnormalities in the CN-AML patients. (**A**) Comparison of the CR rates between the CN-AML patients with normal (*WT1* ^ctr^) or high (*WT1* ^op^) *WT1* gene expression. (**B**) Comparison of the CR rates among three different risk subgroups that were stratified based on molecular abnormalities, *i.e*., the favorable risk group included CN-AML patients that are carrying the *NPM1* ^mt^/no *FLT3* ^ITD^ genotypes; the unfavorable risk group with the *FLT3* ^ITD^ genotypes; and the intermediate group are those patients other than the two other risk groups, *i.e*., lack of the *NPM1* ^mt^/no *FLT3* ^ITD^ and *FLT3* ^ITD^ genotypes [^8^]. (**C**) Possible role of *WT1* overexpression in determining the CR rates among the three risk subgroups. Abbreviations: *WT1* ^ctr^, normal *WT1* expression; *WT1* ^op^, *WT1* overexpression.

To examine the potential role of *WT1* overexpression in predicting treatment outcome of the CN-AML patients, we first evaluated the impact of the *WT1* overexpression on patient’s response to induction chemotherapy. Complete remission (CR) rates were used and calculated for this evaluation. As shown in **Figure 1A**, the CR rate was significantly influenced by the level of *WT1* gene expression [*p = .003*]. While about 76% (56/74) of the CN-AML patients in the normal *WT1* gene control group had complete remission after treatment, close to half of those patients 45% (13/29) with *WT1* overexpression had complete remission.

Since the *WT1* overexpression clearly showed its impact on patient’s response to induction chemotherapy, we next tested the possible role of *WT1* overexpression in measuring CR rates among the 3 different risk groups as defined by the *FLT3* and *NPM1* mutation status. Consistent with the previous classification [2], [3], the CR rates in our patient cohort were positively correlated with the three defined risk molecular subgroups (**Figure 1B**), *i.e*., the CR rates were found to be 91% (21/23), 69% (43/62) and 28% (5/18) in the favorable (*NPM1* ^mt^/no *FLT3* ^ITD^), intermediate and unfavorable (*FLT3* ^ITD^) groups, respectively.

The *WT1* gene expression status was then added to the data analyses and compared for their CR rates among the 3 different risk groups. As shown in **Figure 1C**, it is evident that *WT1* gene expression status had no effect on CR rate in the intermediate risk group. However, statistically significant differences were revealed between patient groups with or without high *WT1* gene expression in the favorable (*NPM1* ^mt^/no *FLT3* ^ITD^) and the unfavorable (*FLT3* ^ITD^) risk groups. For example, in the favorable (*NPM1* ^mt^/no *FLT3* ^ITD^) patient group, complete remission was seen in all 19 patients when the *WT1* gene was expressed at the normal level. However, only half of those patients (2 out of 4) showed complete remission when *WT1* gene was overexpressed (*p = .024*). Remarkably, in the unfavorable (*FLT3* ^ITD^) risk group, none of the patient with *WT1* overexpression showed complete remission whereas about half of this group of patient (5 out of 10) with normal *WT1* expression had complete remission (*p = .036*).

Altogether, the CR rate analyses suggested that *WT1* overexpression alone has no clear role in predicting CR in the intermediate risk group. It however could potentially play a functional role in predicting CR of the CN-AML patients either in the favorable (*NPM1* ^mt^/no *FLT3* ^ITD^) or the unfavorable (*FLT3* ^ITD^) groups. Specifically, when *WT1* overexpression is added to the predefined risk groups, it may increase the risk or abridge the favorable outcome of the CN-AML patients by interactions with the favorable group or with the unfavorable group, respectively.

### Role of *WT1* Overexpression in Predicting Disease-free and Overall Survivals

**Figure 2:**
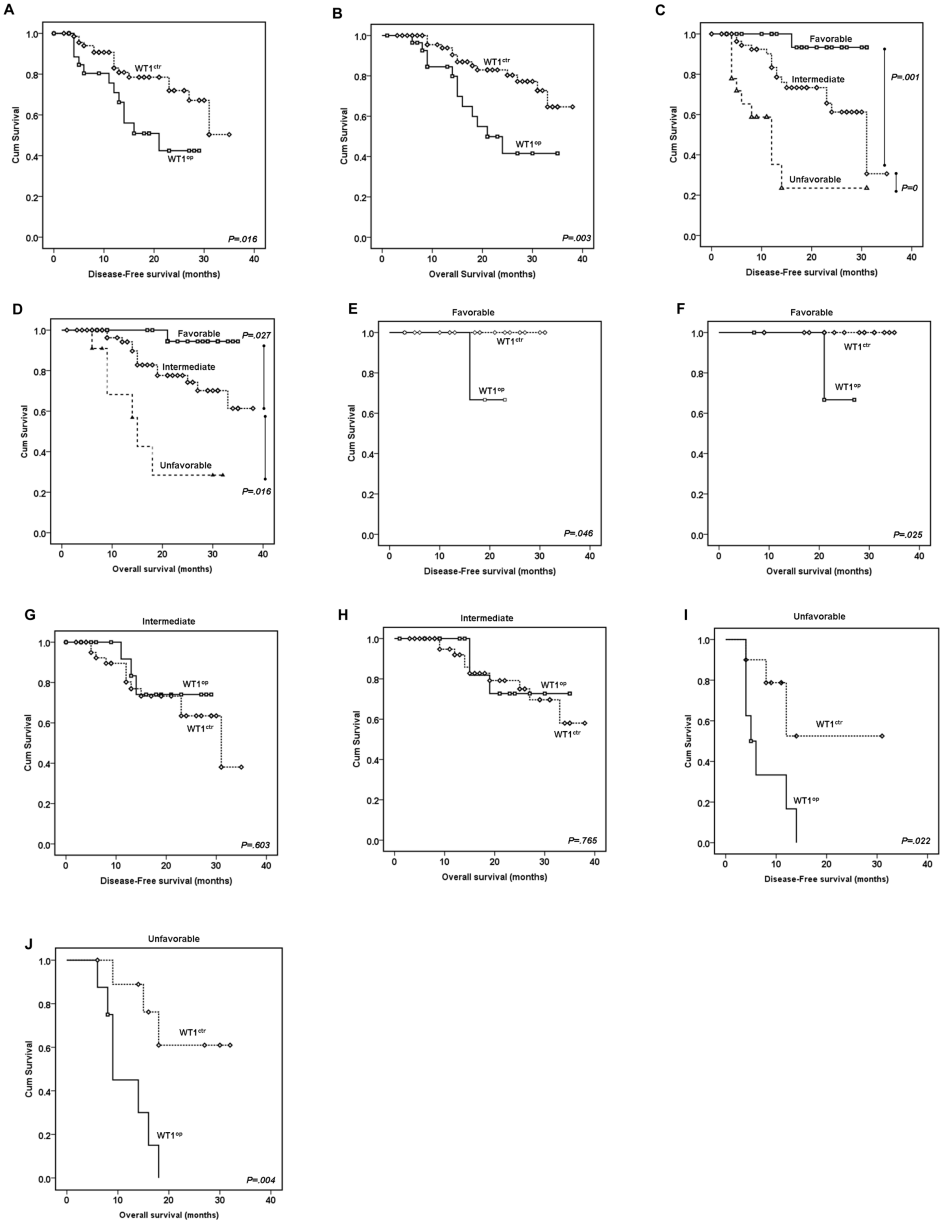
Determination of the diease-free survival (DFS) and overall survival (OS) in the CN-AML patients based on the *WT1* expression status and other molecular abnormalities. (**A–B**) Comparison of the DFS (**A**) and OS (**B**) between the CN-AML patients with normal (*WT1* ^ctr^) or high (*WT1* ^op^) *WT1* gene expression. The mean DFS and OS of patients with *WT1* ^op^ (n = 29) or *WT1* ^ctr^ (n = 74) were 18.9±2.1 *vs*. 27.8±1.4 mo (*p = .016*); and 23.6±2.3 *vs*. 32.5±1.3 mo (*p = .003*), respectively. (**C–D**) Comparison of the DFS (**C**) and OS (**D**) among three different risk subgroups that were stratified based on molecular abnormalities, *i.e*., the favorable risk group included CN-AML patients that are carrying the *NPM1* ^mt/^no *FLT3* ^ITD^ genotypes; the unfavorable risk group with the *FLT3* ^ITD^ genotypes; and the intermediate group are those patients other than the two other groups, *i.e*., lack of the *NPM1* ^mt^/no *FLT3* ^ITD^ and *FLT3* ^ITD^ genotypes [^8^]. The average DFS of the patients with favorable (n = 23), intermediate (n = 62) or unfavorable genotype (n = 18) were 30.0±1.0, 26.6±1.6 and 13.9±3.0 mo, respectively. The average OS of the patients with favorable (n = 23), intermediate (n = 62) or unfavorable genotype (n = 18) were 34.2±0.85, 31.4±1.5 and 19.0±2.5 mo, respectively. (**E–J**) Possible role of *WT1* overexpression in determining the DFS (**E, G and I**) and OS (**F, H, and J**) among the favorable (**E–F**; *NPM1* ^mt^/no *FLT3* ^ITD^), the intermediate (**G–H**) and the unfavorable (**I–J**; *FLT3* ^ITD^) molecular and risk subgroups. Note that patients with *WT1* ^op^ in the favorable (n = 4) or unfavorable (n = 8) had inferior DFS and OS than their control *WT1* ^ctr^ groups (favorable, n = 19; unfavorable, n = 10). No significant differences of DFS and OS were observed between normal and high *WT1* gene expression in the intermediate group. Abbreviations: *WT1* ^ctr^, normal *WT1* expression; *WT1* ^op^, *WT1* overexpression.

Since *WT1* overexpression appeared to play a functional role in predicting complete remission of the CN-AML patients especially when it is combined with the favorable *NPM1* ^mt^/no *FLT3* ^ITD^ or the unfavorable *FLT3* ^ITD^ genotypes, we next examined the potential impact of *WT1* overexpression on the disease-free survival (DFS) and the overall survival (OS) of the studied CN-AML patient cohort. The CN-AML patients were followed in a time period of 1–38 months with a median value of 18 months for DFS or 13 months for OS, respectively. The Kaplan-Meier overall survival analysis was used to calculate the DFS and OS. The potential difference between each testing groups was analyzed by the log-rank test. The final results are summarized in **Figure 2**.

Comparison of the DFS and OS between the *WT1* overexpression group with the *WT1* control group showed that *WT1* overexpression has significantly reduced patient’s DFS (**Figure 2A**; log rank = 5.847, *p = .016*) and the OS (**Figure 2B**; log rank = 8.616, *p = .003*). As comparison references for the effect of *WT1* overexpression, the impact of other single gene mutational effect such as the *FLT3* ^ITD^, *NPM1* ^mt^ or *FLT3* ^TKD^ mutations on DFS and OS was also evaluated. Consistent with the prior report [2], the *FLT3* ^ITD^ genotype played a strong role in predicting DFS and OS (log rank = 20.641, *p = 0* for DFS and log rank = 19.157, *p = 0* for OS). In contrast, little or no significant influence was detected in patients with the *NPM1* ^mt^ genotype (log rank = 2.146, *p = .143* for DFS and log rank = 2.325, *p = .127* for OS); or with the *FLT3* ^TKD^ genotype (log rank = 0.712, *p = .399* for DFS and log rank = 0.176, *p = .675* for OS).

Disease-free survival and the overall survival were also calculated against the three different risk subgroups. Consistent with previous classifications, significant differences in DFS and OS were indeed found among the three risk subgroups. Specifically, patients in the favorable risk group (*NPM1* ^mt^/no *FLT3* ^ITD^) showed excellent DFS and OS; while the high risk or the unfavorable risk (*FLT3* ^ITD^) group displayed very poor outcomes of the DFS and OS (**Figure 2C and 2D**) with the intermediate groups lied in between.

When combining the *WT1* overexpression with the three different groups, a very similar contributing patterns of the *WT1* overexpression, as we saw in calculation of the CR rates, were observed with regard to its interaction with the three different risk subgroups. Specifically, the *WT1* overexpression did not seem to affect the DFS and OS in the intermediate subgroup (**Figure 2G and 2H**; log rank = 0.270, *p = .603* for DFS, log rank = 0.089, *p = .765* for OS). The average survivals of this group of patients with normal WT1 (*WT1* ^ctr^; n = 45) or high WT1 (*WT1* ^op^; n = 17) expression were 25.4±1.9 and 24.8±2.1 mo (*p = .603*) for DFS, and 30.6±1.8 and 29.9±2.5 mo (*p = .765*) for OS, respectively. However, the *WT1* overexpression significantly reduced the DFS and OS in the favorable risk (*NPM1* ^mt^/no *FLT3* ^ITD^) patient group (**Figure 2E and 2F**; log rank = 4, *p = .046* for DFS, log rank = 5; *p = .025* for OS). The average survivals of this group of patients with normal WT1 (*WT1* ^ctr^; n = 19) lived well beyond the study period, however, 7 out the 8 (88%) patients in the high WT1 (*WT1* ^op^; n = 8) group had DFS of less than 16.0 mo (*p = .046*) or OS of about 21.0 mo (*p = .025*), respectively. The other patient was only followed up to 10 months; thus no specific DFS or OS could be assigned at the completion of this study. Most significantly, the *WT1* overexpression appeared to abruptly shorten patent’s survival of the unfavorable risk group patients in both categories (**Figure 2I and 2J**; log rank = 5.246; *p = .022* for DFS, log rank = 8.481; *p = .004* for OS).

Based on these results, we suggest that *WT1* overexpression by itself could play an important but negative role in predicting DFS and OS of the CN-AML patients; moreover, the *WT1* overexpression, when combined with the *NPM1* ^mt^ or the *FLT3* ^ITD^ genotypes, will serve as a poor prognostic marker in reducing DFS and OS in the favorable risk (*NPM1* ^mt^/no *FLT3* ^ITD^) patient group or worsening the patient outcomes in the unfavorable risk (*FLT3* ^ITD^) group.

### Multivariate Analysis of the *WT1* Overexpression and its Role in Prognosis of CN-AML

Here we were interested in further testing whether *WT1* overexpression is an independent prognostic factor in predicting DFS or OS the CN-AML patients. Multivariate analysis was carried out by using the cause-specific Cox regression model to analyze the relationship among age (age ≤60 years *vs*. >60 years), the *WT1* overexpression, other single gene mutations and the three molecular risk subgroups. As shown in **Table 2** that age, *FLT3* ^ITD^ and *NPM1* ^mt^/no *FLT3* ^ITD^ are all independent prognostic markers as previously reported [2], [3]. Significantly and indeed, the *WT1* overexpression also appeared to be an independent prognostic marker for DFS and OS (*p* = .*028*).

**Table 2:** Multivariate analysis (Cox regression) for clinical and molecular variables of DFS and OS in CN-AML patients.

Variant	DFS		OS	
	Hazard ratio (95% CI)	*P*	Hazard ratio (95% CI)	*P*
Age#	0.32 (0.12∼0.87)	.*025* *	0.37 (0.13∼1.07)	.*037* *
WT1^op^	2.17 (0.96∼4.92)	.*034* *	2.50 (1.10∼5.68)	.*028* *
NPM1^mt^	1.68 (0.41∼6.82)	.*470*	0.92 (0.25∼3.40)	*0.9*
FLT3^TKD^	2.71 (0.89∼8.33)	.*082*	2.01 (0.55∼7.28)	.*289*
FLT3^ITD^	3.35 (1.26∼8.92)	.*016* *	3.91 (1.42∼10.72)	.*008* *
**Risk molecular subgroups**				
**Favorable**				
NPM1^mt^/no FLT3^ITD^	0.07 (0.01∼0.88)	.*039* *	0.16 (0.07∼1.01)	.*041* *
**Unfavorable**				
FLT3^ITD^	3.35 (1.26∼8.92)	.*016* *	3.91 (1.42∼10.72)	.*008* *
**Intermediate**				
Others excluding NPM1^mt^/no FLT3^ITD^ and FLT3^ITD^	1.15 (0.25∼5.39)	.*86*	1.27 (0.34∼6.16)	.*81*

CN-AML, cytogenetically normal acute myeloid leukemia; DFS, disease-free survival; OS, overall survival; CI, confidence interval; WT1^op^, WT1 overexpression.

# age ≤60 years vs. >60 years.

*P values <.05.

## Discussion

Chromosomal aberrations have traditionally been used to assess treatment response, prognosis and survival of the AML patients. Subsequent studies showed, however, that about 85% of the CN-AML patients carried one or more molecular mutations [12]. Indeed, characterization of gene mutations such as *FLT3* or *NPM1* further helped to define the clinical outcomes of AML patients especially when these patients present with normal cytogenetics. Therefore, identification of new molecular biomarker and testing its association with the existing biomarkers are of particular importance for better characterization and risk stratification of CN-AML patients and thus for better patient care.

WT1 overexpression has been shown to play a role in hematologic malignancy [5]. However, molecular mechanism of WT1 overexpression in CN-AML remains to be elusive. There are a number of downstream effectors of the *WT1* genes [13]. For example, the heparin-binding growth factor midkine (*MK*) gene is a prognostic biomarker for various cancers [14], [15]. The insulin-like growth factor I receptor (IGF-I-R) is another known downstream effector [16]. More importantly, many of those downstream effectors are indeed involved in cellular growth or survival. Early study by using antisense oligonucleotides has showed that WT1 is required not only for proliferation but also for inhibiting apoptosis in tumor cell cultures [17]. Therefore, *WT1* overexpression could potentially be used as a tumor-specific target for cancer treatment. Intriguing, an early trial by using peptide vaccines against WT1 in leukemia patients did show promising results [18].

In this study, we examined the possible role of *WT1* gene overexpression in CN-AML patient’s responses to induction chemotherapy and in predicting the treatment outcome such as the disease-free survival or overall survival. We further evaluated the possible contribution of *WT1* overexpression to the identification of risk groups that are typically stratified by genotypes such as the *NPM1* or *FLT3* mutation status, which are known molecular markers associated with the survival and treatment outcomes of the AML patients.

From this study, we found that *WT1* overexpression by itself conversely correlated with the CR rate, DFS and OS (**Figure 1** **and** **2**). Furthermore, *WT1* overexpression also contributes, as a negative prognostic marker, to the prognosis and therapeutic response of the CN-AML patients in the favorable (*NPM1* ^mt^/no *FLT3* ^ITD^) and the unfavorable (*FLT3* ^ITD^) molecular subgroups (**Figure 1** **and** **2**). Specifically, based on the observations of this CN-AML patient cohort, patients with the *WT1* overexpression/*FLT3* ^ITD^ genotypes showed the worst CR rate, OS and DFS (*WT1* ^op^ in **Figure 2I and 2J**). Conversely, patients with normal *WT1* expression and the *NPM1* ^mt^/no *FLT3* ^ITD^ genotypes displayed the best outcome (*WT1* ^ctr^ in **Figure 2E and 2F**). Altogether, our data suggest that the *WT1* gene overexpression is an independent and negative prognostic factor in predicting patient’s response to induction chemotherapy and treatment outcomes. In addition, when combined with the *NPM1* ^mt^ or the *FLT3* ^ITD^ genotypes, the *WT1* overexpression also contribute negatively to patient’s response to induction chemotherapy and treatment outcomes. Please note that any clear therapeutic effect of complete remission described here is almost certainly contributed by multifactorial factors such as age, patient’s physical status, peripheral white blood counts among other molecular factors, *e.g*., *NPM1* ^mt^, *FLT3* ^ITD^, *etc*. What we have showed here suggesting *WT1* gene overexpression is at least one of the molecular factors that could play a negative prognostic role in predicting complete remission.

*WT1* plays an important role in pathogenesis of AML, but its specific function remains elusive or controversial [5]. In some of the earlier studies, the *WT1* overexpression at diagnosis was shown to be as an adverse predictor for CR rate, DFS and OS in AML patient. In contrast, other studies suggested that *WT1* overexpression was not associated with disease outcome [19], [20]. Moreover, previous CN-AML studies on the *WT1* overexpression was mainly used as a molecular marker to detect minimal residual disease. Little was known about the prognostic significance of the *WT1* overexpression in CN-AML. Intriguingly, Frederik Damm and co-workers did suggest earlier that *WT1* overexpression could potentially be used as one of the several biomarkers to formulate some kind of integrative prognostic risk score for the stratification of CN-AML [21]. Indeed, our data showed here that the *WT1* overexpression can not only be used as a negative prognostic marker for CN-AML but also, for the first time to the best of our knowledge, contributes to the identification of the CN-AML risk subgroups that are normally stratified by the *NPM1* and *FLT3* mutation status.

It was noticed that the percentage of the *WT1* overexpression showed in our study cohort was somewhat lower than the previous reports (28% *vs*. 48%–73%) [7], [8]. One obvious difference between our study and the other reports was the use of the reference gene in determining the level of *WT1* gene expression. For example, the housekeeping gene *ABL* was used in our study as an internal control for the calculation of the *WT1* gene expression. The cutoff for normal *WT1* expression was defined as 250 copies/10^4^ *ABL* as previously recommended [9]. In contrast, different cutoff value of the *ABL* gene or other housekeep gene such as *GAPDH* gene was used in the other studies [7], [20]. It is also possible that other molecular aberrations, *e.g*., MiR-15a/16-1, which is as a tumor suppressor that down-regulates *WT1* expression in the process of leukemia cell proliferation [22], could be another contributing factor to the observed differences in the *WT1* overexpression. In spite of the observed differences in the percentages of the *WT1* gene expression, this difference should not affect the fact that *WT1*, when it is overexpressed, contributes negatively to the pathogenesis of CN-AML.

Combining with all of our data, our results strongly suggest that *WT1* overexpression is an independent and negative prognostic biomarker that could potentially be used to evaluate response to induction chemotherapy and prognosis of AML patients with normal cytogenetics. In addition, the use of *WT1* overexpression as an additional biomarker seems to enhance the statistical power in the identification of risk subgroups that are normally stratified solely based on the *NPM1* or the *FLT3* mutational status. Therefore, adding profiling of *WT1* gene expression level in the future decision-making of patient’s response to induction chemotherapy or prognosis of the CN-AML patients could potentially provide better or more effective care of this group of patients.
